# An association between membranoproliferative glomerulonephritis and metastatic colorectal carcinoma: a case report

**DOI:** 10.1186/s13256-016-0979-3

**Published:** 2016-07-20

**Authors:** Samuel Chan, Kimberley A. Oliver, Nicholas A. Gray

**Affiliations:** 1 Department of Renal Medicine, Nambour General Hospital, Nambour, Queensland Australia; 2 The University of Queensland, School of Medicine, Brisbane, Queensland Australia; 3 Department of Pathology, Princess Alexandra Hospital, Woolloongabba, Queensland Australia; 4 The University of Queensland, Sunshine Coast Clinical School, Nambour General Hospital, Nambour, Queensland Australia

**Keywords:** Carcinoma, Colorectal, Glomerulonephritis, Membranoproliferative, Nephrotic syndrome

## Abstract

**Background:**

Membranoproliferative glomerulonephritis is a common pattern of glomerular injury in monoclonal gammopathy, but has only rarely been associated with solid organ tumors, mainly lung, renal, gastric, breast, and prostate. There have been two reported cases of membranoproliferative glomerulonephritis associated with adenocarcinoma of the colon and rectum, although the association may be coincidental. We report a case where nephrotic syndrome due to membranoproliferative glomerulonephritis developed in a patient with colorectal carcinoma and elucidate some of the pathophysiological mechanisms underpinning this presentation.

**Case presentation:**

A 54-year-old white man with a history of adenocarcinoma of the colon with metastasis to the liver and ureter presented with a 1-week history of bilateral pedal edema, and worsening hypertension and renal function. A renal biopsy confirmed membranoproliferative glomerulonephritis type I. Curative therapy for the malignancy was not possible, so treatment was commenced with prednisolone with consequential biochemical improvement in renal function and proteinuria, although his serum albumin remained low.

**Conclusions:**

This case report illustrates an association between membranoproliferative glomerulonephritis and metastatic colorectal carcinoma and adds to the evidence to consider malignancy to be an underlying pathology among newly diagnosed cases of nephrotic syndrome. In the clinical setting, treatment of the underlying malignancy should be first considered in patients with a tumor presenting with kidney disease which is suspected to be paraneoplastic in etiology.

**Electronic supplementary material:**

The online version of this article (doi:10.1186/s13256-016-0979-3) contains supplementary material, which is available to authorized users.

## Background

Nephrotic syndrome can be associated with various solid organ tumors and lymphoproliferative diseases [1–3]. Membranous glomerulonephritis is the most common underlying etiology [3, 4]. Membranoproliferative glomerulonephritis (MPGN) is a common pattern of glomerular injury in monoclonal gammopathy [4], but has only rarely been associated with solid organ tumors, mainly lung [5, 6], renal [5, 7], gastric [4], breast [4], and prostate [5, 8]. There have been two reported cases of MPGN associated with adenocarcinoma of the colon and rectum [5, 9], although the association may be coincidental. We report a case where MPGN developed in the setting of chemotherapy for metastatic colorectal cancer.

## Case presentation

A 54-year-old white man was referred with a 1-week history of bilateral pedal edema, worsening hypertension, and a rise in serum creatinine from 111 μmol/L to 213 μmol/L over 2 weeks. He had microscopic hematuria and proteinuria quantified at 24 grams/day (see Additional file 1). His serum albumin was 24 g/L compared with 35 g/L 2 weeks earlier. Other relevant investigations included negative hepatitis B and C serology, weakly positive speckled antinuclear antibody (ANA) titer of 160 (normal <40) with double-stranded deoxyribonucleic acid (DNA) of 0 IU/mL (normal <7), normal ratio of serum free light chains, negative cryoglobulin screen, negative serum protein electrophoresis, and a normal C3 of 1.77 g/L and C4 of 0.38 g/L.

He had a background history of hypertension, obstructive sleep apnea, and a 3-year history of colorectal carcinoma with hepatic metastasis. His initial treatment included neoadjuvant chemotherapy with capecitabine and bevacizumab, and 26 fractions of radiotherapy. He then underwent a right hemicolectomy and a partial right hemihepatectomy, followed by 6 months of capecitabine and bevacizumab. One year later, a new 74 mm metastatic lesion developed in his residual right liver lobe, and a 16 mm mucin-secreting adenocarcinoma occurred at the left vesicoureteric junction, which was managed by distal ureterectomy. He was recommenced on capecitabine, bevacizumab, and cetuximab, and continued on this treatment until presentation with nephrotic syndrome.

**Fig. 1:**
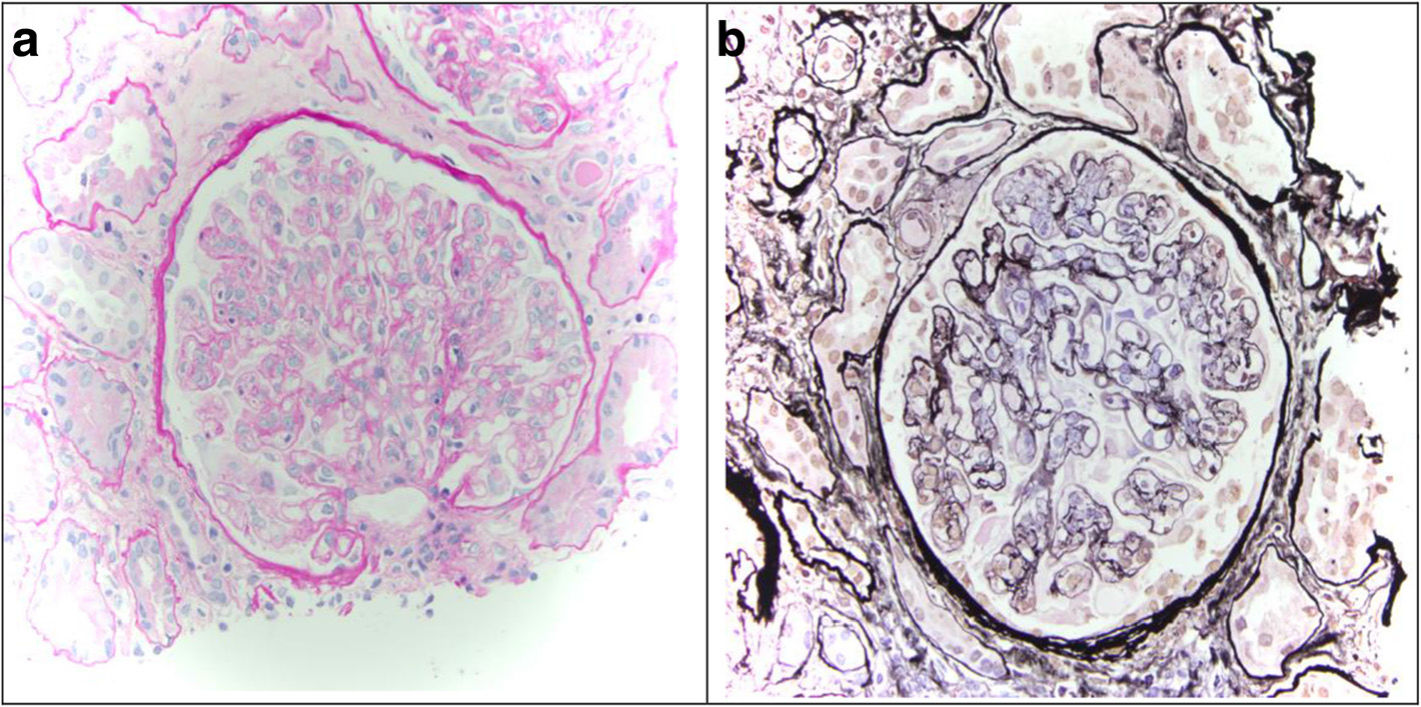
**a** Periodic acid–Schiff stain, 100× magnification, showing features of membranoproliferative glomerulonephritis type 1. **b** Methenamine silver stain, 200× magnification, highlighting double contour formation in capillary loops. Mesangial proliferation and segmental endocapillary proliferation can also be seen

A renal biopsy was diagnostic of MPGN type I. Functioning glomeruli showed mesangial hypercellularity, endocapillary proliferation, and double contours in capillary loops. No hyaline deposits were noted in the capillary loops and no segmental sclerosis was seen (Fig. 1a). There was interstitial fibrosis and tubular atrophy together with lymphocytes, plasma cells, and eosinophils in the scarred interstitium (Fig. 1b). Immunofluorescence showed moderate granular deposition of IgG and C3 in the mesangial areas and around the capillary loops. Electron microscopy showed deposits in the mesangial, paramesangial, and subendothelial regions. Focal duplication of the glomerular basement membrane was seen and there was mild expansion of mesangial matrix.

Curative therapy for the malignancy was not possible, so treatment was commenced with prednisolone for the renal disease. Four months later, his proteinuria had reduced to 1.4 grams/day, creatinine had improved to 155 μmol/L, and albumin returned to baseline of 31 g/L.

## Discussion

This case highlights that MPGN can be associated with malignancy, although the mechanism underpinning this association has not been completely elucidated.

Little is known about the association between MPGN and malignancy, although there is a wealth of published information regarding nephrotic syndrome and malignancy. As early as the 1960s, there were suggestions that nephrotic syndrome may be related to thyroid cancers, Hodgkin’s lymphoma, or chronic lymphocytic leukemia [1]. Further, a more recent study analyzed approximately 8 million people in the Danish National Registry of Patients, estimating that that 4.7 % of patients who had nephrotic syndrome (from a total of 4293 patients) had underlying malignancy with lung cancer, multiple myeloma, and prostate cancer being most likely [10].

There are two main hypotheses which have been proposed to explain the pathogenesis of the association between MPGN and malignancy. First, an undiagnosed malignancy associated with immune complex deposition may cause a glomerular disease-like paraneoplastic syndrome. While data are limited in MPGN, studies have shown that paraneoplastic membranous nephropathy is characterized by an increased number of inflammatory cells in the glomeruli compared with that of idiopathic membranous nephropathy [4, 11]. There is a greater prominence of the subtypes of IgG1 and IgG2 in the renal biopsy samples of patients with paraneoplastic membranous nephropathy compared with idiopathic membranous nephropathy [12]. Second, viral infection may have induced both glomerulopathy and cancer by intrinsic viral oncogenic activity. It is possible that disrupted renal clearance of biological mediators may be associated with oncogenesis [13]. However, the most likely explanation for the connection between virus latency and tumorigenesis is that replicating viruses may initiate cell death [14]. When latent viruses produce virions, virus replication generates pathogen-associated molecular patterns from partially synthesized viral chromosomes, double-stranded ribonucleic acid (RNAs) and empty capsids that trigger cellular DNA damage responses and innate immune signaling. Toll-like receptor and interferon signaling by virus infection is subsequently activated, amplifying the innate response [14].

Remission of cancer has led to subsequent remission of nephrotic syndrome. Reports have shown that patients who have paraneoplastic glomerulopathy secondary to renal cell carcinoma have undergone remission or a reduction of proteinuria through either radical nephrectomy, nephron-sparing, partial nephrectomy, or laparoscopic nephrectomy [15]. There are, however, no reports that have specifically looked at the remission of cancer in patients with MPGN. There is some evidence for the use of prednisolone when cure of the cancer is not an option. One case of MPGN developed after surgical removal of a bronchial carcinoid tumor, but responded to prednisolone therapy [16]. Furthermore, there has been a case showing use of prednisolone for paraneoplastic MPGN without ablation in a patient with metastatic prostate cancer [7]. Moreover, paraneoplastic glomerulopathy developed in a patient with minimal change disease, who had retroperitoneal sarcoma, probably secondary to a T-cell-mediated response to malignancy, which went into remission with corticosteroid therapy [17]. Administration of corticosteroids was considered in this setting primarily because of the patient’s high performance status.

The prognosis of MPGN in the setting of malignancy is uncertain [18]. The presence of hypertension at presentation, severe nephrotic syndrome, and renal insufficiency are known poor prognostic factors among non-malignancy-associated nephrotic syndrome [18].

## Conclusions

In summary, we report a case of an association between MPGN and metastatic colorectal cancer. Clinicians need to be vigilant about the possible paraneoplastic nature of MPGN. Initial treatment of the underlying malignancy should be the primary management in patients presenting with kidney disease which is suspected to be paraneoplastic in etiology, although prednisolone is an appropriate therapy if cure of the cancer is not feasible.

## Additional file

Additional file 1: Urine protein evidence. (PDF 105 kb)
