# Supplementary material for: An association between membranoproliferative glomerulonephritis and metastatic colorectal carcinoma: a case report
Source: J Med Case Rep. 2016 Jul 20;10:199. doi: 10.1186/s13256-016-0979-3 (PMC4955154; doi:10.1186/s13256-016-0979-3)
Supplement: Additional file 1: — Urine protein evidence. (PDF 105 kb) [file 13256_2016_979_MOESM1_ESM.pdf]

Lab No / Specimen Type	681362775 Urine ? Collection	681367680 Urine 24h Collection	701440726 Urine	701560656 Urine ? Collection	**701565101 Urine 24h Collection**	Units	Ref Range
**Concentrations**							
Creatinine	8.9	6.8	6.6	5.0	6.6	mmol/L	
Protein	**1300**	**11000**	**6500**	**2200**	**1800**	mg/L	(< 100)
**Timed Excretion**							
Time Period		24.0			24.0	h	
Volume		2.196			1.956	L	
Creatinine		14.9			12.9	mmol/24h	(8.0 - 18.0)
Protein		**24000**			**3500**	mg/24h	(< 150)
**Creatinine Ratios**							
Protein	**146**	**1600**	**985**	**440**	**269**	g/mol creat	(< 15)
